# AngioJet rheolytic thrombectomy in patients with thrombolysis in myocardial infarction thrombus grade 5: an observational study

**DOI:** 10.1038/s41598-022-09507-z

**Published:** 2022-03-31

**Authors:** Yi-xiong Huang, Yi Cao, Yu Chen, Yi-gang Qiu, Jian-yong Zheng, Ying-ming Liu, Jiang-chun He, Li Zhao, Tian-chang Li

**Affiliations:** 1 Medical School of Chinese People’s Liberation, Army General Hospital, 28 Fuxing Road, Haidian District, Peking, 100853 People’s Republic of China; 2 Department of Cardiology, Sixth Medical Center of Chinese People’s Liberation, Army General Hospital, 6 Fucheng Road, Haidian District, Peking, 100048 People’s Republic of China

**Keywords:** Cardiac device therapy, Interventional cardiology

## Abstract

The aim of this study was to evaluate the effectiveness and safety of AngioJet rheolytic thrombectomy among patients with high thrombus burden. Routine manual thrombus aspiration in patients with ST-segment elevation myocardial infarction (STEMI) does not improve clinical outcomes and was associated with an increased rate of stroke. However, the safety of mechanical thrombus aspiration is still unknown. This was a retrospective, single-center study involving 621 patients with Thrombolysis In Myocardial Infarction thrombus grade 5. The primary outcome was the composite of major adverse cardiovascular events (MACE) within 12 months. The safety outcome was stroke within 1-year. Propensity matching score was calculated due to the significant baseline differences between the AngioJet rhelytic thrombectomy group and the routine treatment group. AngioJet rheolytic thrombectomy was performed in 117 patients. After propensity-score matching, there was no significant difference both in the incidence of MACE (11.1% vs 17.9%, hazard ratio, 1.641; 95% confidence interval [CI] 0.822 to 3.277, p = 0.161) and the incidences of stroke (1.7% vs 2.6%, hazard ratio 1.522; 95% confidence interval [CI] 0.254 to 9.107, p = 0.646) between two groups at 1-year follow-up. In patients with Thrombolysis In Myocardial Infarction thrombus grade 5, AngioJet rheolytic thrombectomy did not improve clinical outcomes at 1 year. However, AngioJet rheolytic thrombectomy did not increase the risk of stroke in patients with high thrombus burden.

## Introduction

High thrombus burden is a predicting factor of poor prognosis in patients with ST-Segment elevation myocardial infarction (STEMI)^1,2^. Previous studies failed to show the benefit of routine thrombus aspiration in patients with STEMI by using manual aspiration catheter^3–5^. Similar result was observed in the subgroup of high thrombus burden, which also showed an increased rate of stroke at 1 year in routine thrombus aspiration group^6^. Accordingly, routine use of thrombus aspiration is not recommended in the guidelines for the management of acute myocardial infarction base on the evidence of TALOR study^7^. However, the effectiveness of manual thrombus aspiration is susceptible to operating techniques. The increased risk of stroke in the manual thrombus aspiration group might be pertinent to the detachment of thrombus from aspiration catheter^8^. Mechanical thrombus aspiration is accomplished with high velocity saline jets leading to a low-pressure zone (Bernoulli effect), which is more effective in thrombus removal than manual aspiration. Thus, mechanical removal of thrombus among patients with STEMI may be associated with improved outcomes via avoiding migrant thrombus. Moreover, the selection of suitable candidates and the best time for applying thrombus aspiration are still controversial. In this study, we sought to evaluate the effectiveness and safety of AngioJet rheolytic thrombectomy in patients with Myocardial Infarction Thrombus Grade 5 (total occlusion caused by thrombus before wire crossing)^9^.

## Materials and methods

### Study design and population

This is an observational, retrospective, single-center study. Patients with high thrombus burden (Thrombolysis In Myocardial Infarction thrombus grade 5) admitted to the Sixth Medical Center of Chinese People's Liberation Army General Hospital from October 2014 to April 2019 were enrolled consecutively. All participants met the diagnostic criteria of STEMI according to the universal definition of myocardial infarction^10^: detection of a rise of cardiac troponin with at least one value above the 99th percentile upper reference limit and with at least one of following: symptoms of ischemia; new or presumed new significant ST-segment-T wave changes or new left bundle branch block; development of pathological Q waves in the electrocardiogram; imaging evidence of new loss of viable myocardium or new regional wall motion abnormality. Exclusion criteria included an age younger than 18 years, a rejection of invasive surgery, a contraindication of PCI, severe tortuous and calcified lesion, vessel diameter of culprit lesion less than 2.5 mm, the need for emergency coronary artery bypass.

Ethics Committee of the Sixth Medical Center of Chinese People's Liberation Army General Hospital specially approved that no informed consent was required because data were going to be analyzed anonymously.

### Cardiac catheterisation and coronary intervention

All patients received dual antiplatelet therapy (aspirin 300 mg loading dose and a P2Y_12_ receptor inhibitor: clopidogrel 600 mg loading dose or ticagrelor 180 mg loading dose) before coronary angiography. The use of glycoprotein IIb/IIIa inhibitors or low molecular weight heparin was left to the discretion of the operator. An intra-aortic balloon pump (IABP) was used or not based on the patient’s hemodynamic status. None of the patients underwent manual thrombus aspiration because our center was not equipped with manual aspiration catheter.

The use of AngioJet rhelytic thrombectomy was left at the interventional cardiologist’s discretion. The AngioJet Ultra thrombectomy system (Boston Scientific) consists of a 4-F aspiration catheter, a pump set, and a drive unit console. A brief procedure was as follows. First, prime the catheter by heparinized saline until the time display reaches zero seconds. Activate the device at least 1 cm proximal to the thrombus before advancing beyond the lesion with advancement speed of 2 mm per second, and keep the device activated during multiple passages until all visible thrombus disappears. Single suction time should be less than 5 s to avoid transient bradycardia. Repeat suction should be deployed when TIMI flow did not restore or residual thrombus was detected by angiography.

### Study end point

The primary outcome was the composite of major adverse cardiovascular events (MACE) including death, heart failure, rehospitalization for unstable angina, and target vessel revascularization within 12 months. The safety outcome was stroke within 1-year. Follow-up visits were required for all patients at the time of 1 month, 3 months, 6 months, and 12 months after angiography. Patients living in remote area were followed by phone calls.

### Statistical analysis

The data were analyzed by SPSS version 26.0 for window (SPSS Inc., Chicago, IL, USA). The normality of distribution of continuous variables was tested by one-sample Kolmogorov–Smirnov test. Continuous variables with normal distribution were presented as mean (standard deviation [SD]); non-normal variables were reported as median (interquartile range [IQR]). The frequencies of categorical variables were compared using Pearson χ^2^ or Fisher’s exact test.

Due to the significant baseline differences between two groups, the propensity-score matching method was used. Matching was performed with the use of a 1:1 matching protocol without replacement (greedy-matching algorithm), with a caliper width equal to 0.01 of the standard deviation of the logic of the propensity score.

The time to death within 1 year after angiography according to study group is presented in a Kaplan–Meier plot. Hazard ratios for the primary end point and for all end points assessed through 1 year were calculated with the use of a Cox proportional-hazards model with treatment as the only factor and were shown with the nominal 95% confidence interval from the Cox model and the nominal two-sided p value from a log-rank test. All tests were 2-sided and a p < 0.05 was considered significant.

The authors confirm that all methods were carried out in accordance with relevant guidelines and regulations. The authors confirm that all experimental protocols were approved by Ethics Committee of the Sixth Medical Center of Chinese People's Liberation Army General Hospital.

## Results

### Baseline characteristic

**Figure 1:**
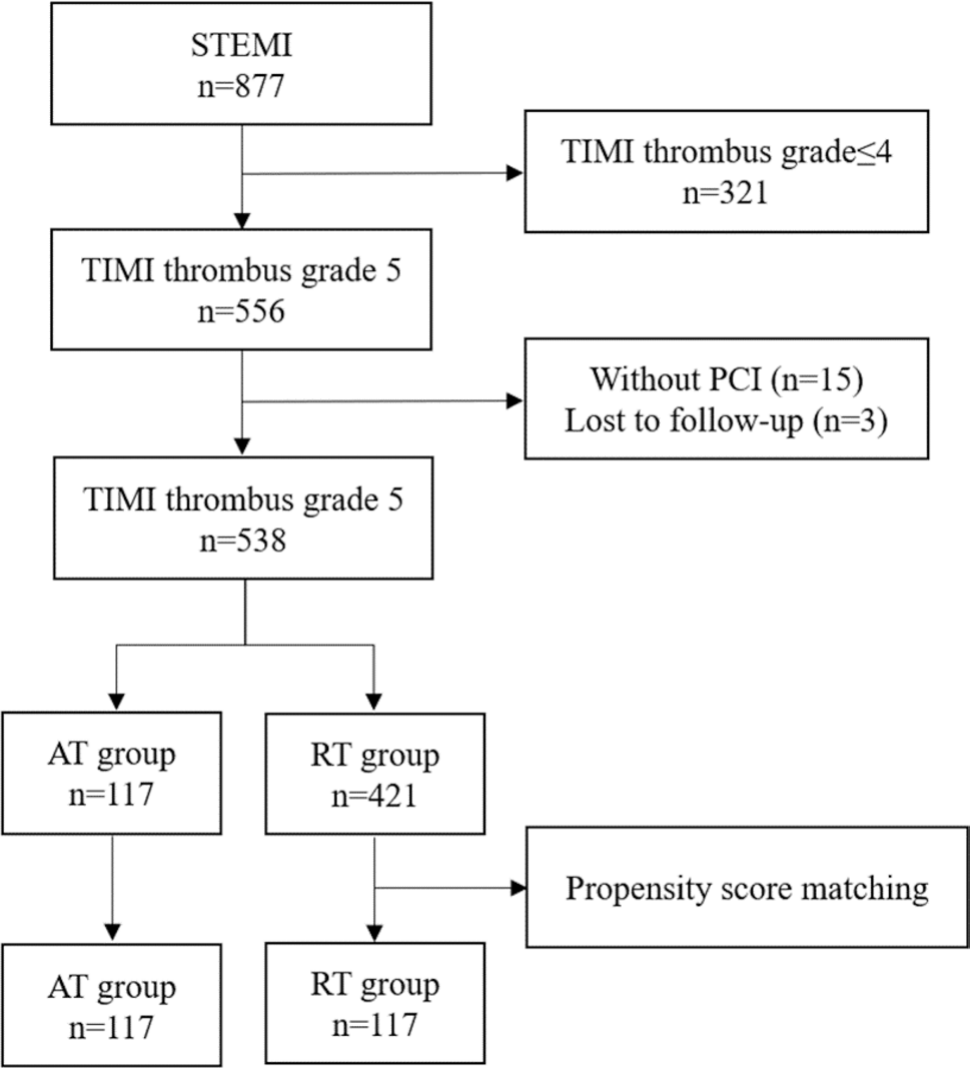
Study population. *STEMI* ST-segment elevation myocardial infarction, *TIMI* thrombolysis in myocardial infarction, *PCI* percutaneous coronary intervention, *AT* AngioJet rheolytic thrombectomy, *RT* routine treatment.

There were 877 consecutive patients diagnosed with STEMI from October 2014 to April 2019. Five hundred and fifty-six of these with TIMI thrombus grade 5 (total occlusion caused by thrombus before wire crossing) were enrolled. Fifteen patients who did not perform PCI were excluded. Three cases were lost to follow-up. Enrolled patients were divided into 2 groups: AngioJet rheolytic thrombectomy group (AT, n = 117), routine treatment group (RT, n = 439). After propensity-score matching, 117 patients in each group were included in the analysis (Fig. 1).

Baseline clinical characteristics of patients before and after propensity-score matching are listed in Table 1. The AT group was younger and had more men than the RT group. There were less delayed PCI and IABP use in the AT group as compared with the RT group. Systolic blood pressure was lower in the AT group at admission. In-hospital data were shown in Table 2. The AT group had higher cardiac troponin I both at admission and peak value as compared with the RT group. Hemoglobin and estimated glomerular filtration rate were significantly higher in the AT group, while brain natriuretic peptide was significantly higher in the RT group. P2Y_12_ inhibitor, statin, β-blocker, and glycoprotein IIb/IIIa inhibitor were used more often in the AT group.

**Table 1:** Baseline characteristics before and after propensity-score matching.

Characteristic	Before matching			After matching		
	AT group (n = 117)	RT group (n = 421)	p value	AT group (n = 117)	RT group (n = 117)	p value
Age, year	60.9 ± 13.9	64.2 ± 13.4	0.019	60.9 ± 13.9	61.1 ± 13.4	0.910
Male (%)	102 (87.2)	322 (76.5)	0.012	102 (87.2)	102 (87.2)	1.000
BMI (kg/m^2^)	25.4 ± 3.52	25.3 ± 3.37	0.759	25.4 ± 3.52	25.9 ± 3.39	0.232
Diabetes mellitus (%)	28 (23.9)	127 (30.2)	0.188	28 (23.9)	29 (24.8)	0.879
Hypertension (%)	63 (53.8)	222 (52.7)	0.831	63 (53.8)	48 (41.0)	0.050
Dyslipidemia (%)	20 (17.1)	58 (13.8)	0.367	20 (17.1)	19 (16.2)	0.861
Current smoking (%)	66 (56.4)	200 (47.5)	0.088	66 (56.4)	64 (54.7)	0.792
Previous MI (%)	7 (6.0)	34 (8.1)	0.450	7 (6.0)	4 (3.4)	0.354
Previous PCI (%)	8 (6.8)	37 (8.8)	0.500	8 (6.8)	6 (5.1)	0.581
Previous stroke (%)	17 (14.5)	56 (13.3)	0.731	17 (14.5)	14 (12.0)	0.563
Killip class ≥ II(%)	16 (13.7)	91 (21.6)	0.057	16 (13.7)	16 (13.7)	1.000
Delayed PCI (%)^a^	3 (2.6)	62 (14.7)	0.000	3 (2.6)	5 (4.3)	0.722
IABP (%)	4 (3.4)	52 (12.4)	0.005	4 (3.4)	5 (4.3)	1.000
Number of stents	1.27 ± 0.60	1.32 ± 0.75	0.550	1.27 ± 0.60	1.29 ± 0.78	0.850
SBP at admission (mmHg)	117.4 ± 19.5	122.4 ± 21.7	0.024	117.4 ± 19.5	122.8 ± 23.0	0.054
DBP at admission (mmHg)	71.5 ± 12.4	73.2 ± 13.6	0.204	71.5 ± 12.4	73.1 ± 14.5	0.354

*BMI* body mass index, *MI* myocardial infarction, *PCI* percutaneous coronary intervention, *IABP* Intra aortic balloon pump, *SBP* systolic blood pressure, *DBP* diastolic blood pressure.

^a^Delayed PCI: the time of PCI from symptom onset > 12 h.

**Table 2:** In-hospital data before and after Propensity-Score Matching.

Characteristic	Before matching			After matching		
	AT group (n = 117)	RT group (n = 421)	p value	AT group (n = 117)	RT group (n = 117)	p value
cTnI at admission (ng/mL)	39.1 (10.1, 85.8)	26.0 (5.4, 50)	0.010	39.1 (10.1, 85.8)	48.2 (6.8, 88)	0.724
cTnI peak (ng/mL)	54.5 (26.9, 88)	45.8 (13.2, 66.8)	0.000	54.5 (26.9, 88)	65.0 (23.1, 107.8)	0.602
CK-MB at admission (ng/mL)	185.8 (73.8, 291.9)	133.9 (40.3, 279.9)	0.044	185.8 (73.8, 291.9)	126.7 (42.7, 241.1)	0.031
CK-MB peak (ng/mL)	200.1 (105.2, 336.9)	173 (57.9, 338.7)	0.124	200.1 (105.2, 336.9)	161.1 (60.2, 273.1)	0.034
Hemoglobin (g/L)	140.6 ± 14.5	135.4 ± 21.0	0.003	140.6 ± 14.5	139.5 ± 19.3	0.631
eGFR (mL/min/1.73 m^2^)	81.6 ± 22.5	76.6 ± 22.6	0.036	81.6 ± 22.5	82.9 ± 20.3	0.629
TC (mmol/L)	4.77 ± 1.16	4.70 ± 1.21	0.577	4.77 ± 1.16	4.92 ± 1.34	0.345
HDL (mmol/L)	1.21 ± 0.33	1.20 ± 0.32	0.815	1.21 ± 0.33	1.23 ± 0.30	0.630
LDL (mmol/L)	2.59 ± 0.78	2.60 ± 0.87	0.933	2.59 ± 0.78	2.56 ± 0.82	0.718
INR	1.14 ± 0.38	1.18 ± 0.55	0.440	1.14 ± 0.38	1.12 ± 0.27	0.654
BNP (pg/mL)	109 (40.8, 276.3)	159.5 (70.3, 472)	0.001	109 (40.8, 276.3)	137.0 (68.8, 346.5)	0.032
Aspirin (%)	114 (97.4)	388 (92.2)	0.043	114 (97.4)	113 (96.6)	1.000
P2Y_12_ inhibitor (%)	114 (97.4)	386 (91.7)	0.032	114 (97.4)	111 (94.9)	0.499
PPI (%)	69 (59.0)	252 (60.0)	0.841	69 (59.0)	63 (53.8)	0.429
Statin (%)	114 (97.4)	387 (91.9)	0.037	114 (97.4)	111 (94.9)	0.499
ACEi or ARB (%)	81 (69.2)	272 (64.6)	0.352	81 (69.2)	78 (66.7)	0.674
β-blocker (%)	91 (77.8)	286 (67.9)	0.040	91 (77.8)	76 (65.0)	0.030
MRA (%)	17 (14.5)	74 (17.6)	0.437	17 (14.5)	15 (12.8)	0.704
Low molecular heparin (%)	4 (3.4)	15 (3.6)	0.940	4 (3.4)	3 (2.6)	1.000
GP IIb/IIIa inhibitor (%)	89 (76.1)	218 (51.8)	0.000	89 (76.1)	94 (80.3)	0.429
LVEF (%)	52.9 ± 8.84	53.5 ± 8.60	0.510	52.9 ± 8.84	53.6 ± 9.19	0.567
TIMI flow grade 3 after PCI	105 (89.7)	366 (86.9)	0.416	105 (89.7)	104 (88.9)	0.832
Lengh of stay (days)	10.6 ± 4.01	11.7 ± 6.39	0.020	10.6 ± 4.01	10.9 ± 5.95	0.616

*cTnI* cardiac troponin I (reference 0–0.04 ng/mL), *CK-MB* creatine kinase-MB, *eGFR* estimated glomerular filtration rate, *TC* total cholesterol, *TG* triglyceride, *HDL* high-density lipoprotein, *LDL* low-density lipoprotein, *INR* international normalized ratio, *BNP* Brain natriuretic peptide, *PPI* proton pump inhibitor, *ACEi* angiotensin-converting enzyme inhibitor, *ARB* angiotensin receptor, *MRA* mineralocorticoid receptor antagonist, *GP* glycoprotein, *LVEF* left ventricular ejection fraction, *TIMI* Thrombolysis in Myocardial Infarction.

The variables used for matching included gender, age, body mass index, history of diabetes mellitus, history of hypertension, history of dyslipidemia, current smoking, previous myocardial infarction, previous PCI, killip class, delayed PCI, IABP, the use of GP IIb/IIIa inhibitor. After propensity-score matching, there were fewer differences in the baseline clinical and interventional features. Creatine kinase-MB at admission was higher in the AT group compared with the RT group. However, there was no significant difference in the peak value of creatine kinase-MB. The triglyceride level was significantly lower in the AT group. There was still more β-blocker used in the AT group after propensity-score matching. No statistical differences were found for the rate of TIMI blood flow grade 3 after PCI between two groups, neither before nor after propensity-score matching.

### Clinical outcomes

Clinical outcomes of the 1-year follow-up before and after propensity matching are shown in Table 3. By 1 year follow-up, the rate of MACE was significantly lower in the AT group as compared with the RT group (11.1% vs 21.6%, hazard ratio, 0.483; 95% confidence interval [CI] 0.270 to 0.863, p = 0.014). The difference was driven by all-cause mortality (4.3% vs 12.8%, hazard ratio 3.127; 95% confidence interval [CI] 1.251 to 7.818, p = 0.015). There was no significant difference in the incidence of stroke between two groups at 1-year follow-up in all patients (1.7% vs 1.9%, hazard ratio 1.201; 95% confidence interval [CI] 0.255 to 5.656, p = 0.817).

**Table 3:** Clinical outcomes at 1 year before and after Propensity-Score Matching.

	Before matching			After matching		
	AT group (n = 117)	RT group (n = 421)	p value	AT group (n = 117)	RT group (n = 117)	p value
**MACE (%)**	13 (11.1)	91 (21.6)	0.014	13 (11.1)	21 (17.9)	0.161
Death (%)	5 (4.3)	54 (12.8)	0.015	5 (4.3)	8 (6.8)	0.398
Heart failure (%)	5 (4.3)	21 (5.0)	0.621	5 (4.3)	6 (5.1)	0.741
Rehospitalization (%)	2 (1.7)	13 (3.1)	0.369	2 (1.7)	5 (4.3)	0.264
TVR (%)	2 (1.7)	8 (1.9)	0.811	2 (1.7)	3 (2.6)	0.643
Stroke (%)	2 (1.7)	8 (1.9)	0.817	2 (1.7)	3 (2.6)	0.646

*TVR* Target vessel revascularization.

**Figure 2:**
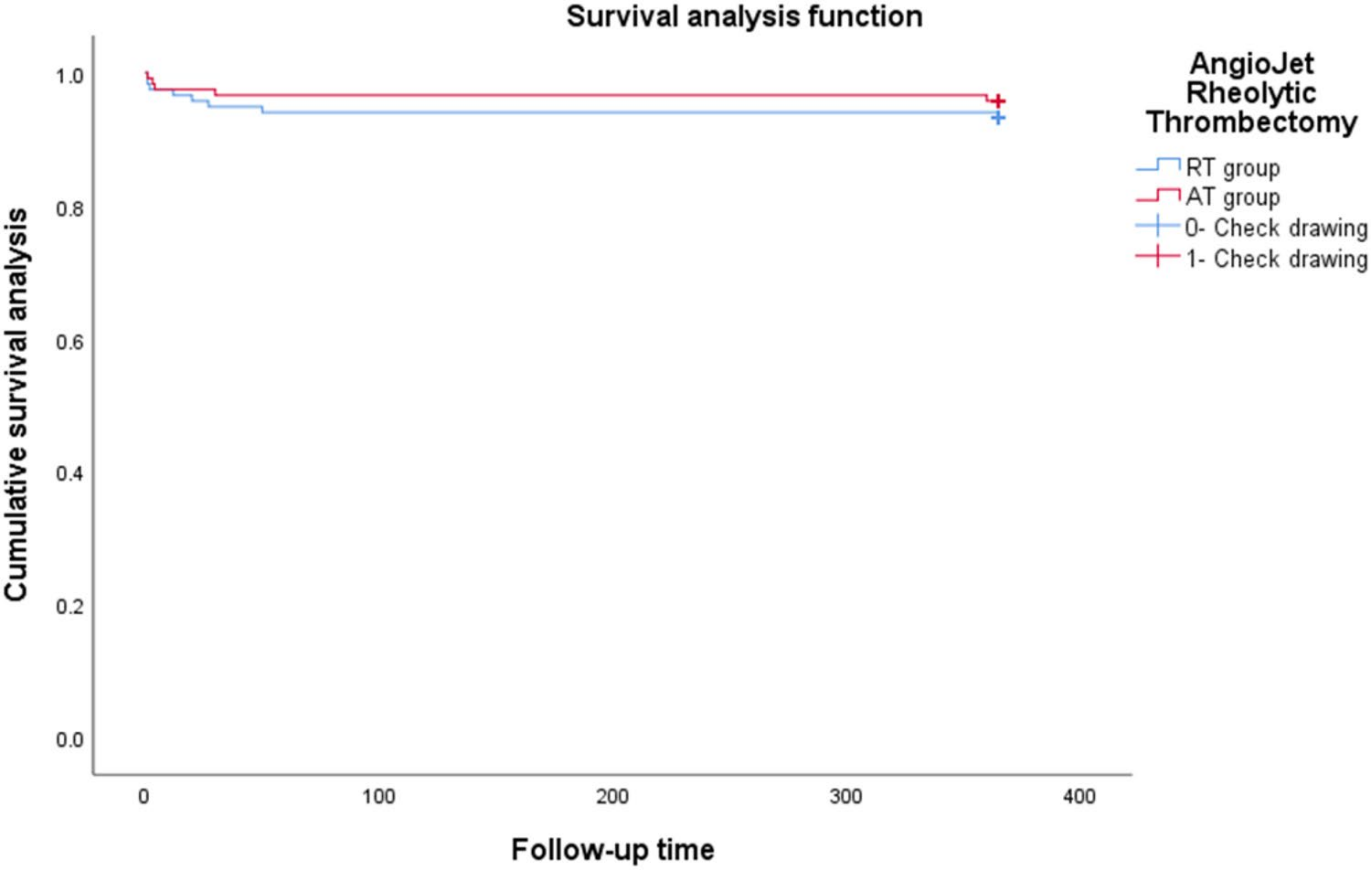
Kaplan–Meier curves for death from any cause.

After propensity-score matching, there was no significant difference in the incidence of MACE between two groups (11.1% vs 17.9%, hazard ratio 1.641; 95% confidence interval [CI] 0.822 to 3.277, p = 0.161). Although the death rate was lower in the AT group as compared with RT group, the difference was not significant (4.3% vs 6.8%, hazard ratio 1.619; 95% confidence interval [CI] 0.530 to 4.948, p = 0.398). Kaplan–Meier Curves for Death from Any Cause are showed in the Fig. 2. The incidence of stroke was still not significant difference between two groups after propensity-score matching (1.7% vs 2.6%, hazard ratio 1.522; 95% confidence interval [CI] 0.254 to 9.107, p = 0.646). Additionally, there was no significant effect of AngioJet Rheolytic thrombectomy on heart failure, rehospitalization, and target vessel revascularization before and after propensity-score matching.

## Discussion

The main finding of our study is that AngioJet rheolytic thrombectomy with PCI in patients with TIMI thrombus grade 5 did not reduce all-cause mortality, heart failure, rehospitalization, target vessel revascularization as compared with routine treatment at 1-year follow-up. However, the AngioJet rheolytic thrombectomy did not increase the risk of stroke in patients with high thrombus burden.

High thrombus burden is associated with poor myocardial perfusion and adverse clinical outcomes^6,11^. An individual patient Meta-Analysis showed that manual thrombus aspiration was associated with fewer cardiovascular deaths (170 [2.5%] versus 205 [3.1%]; hazard ratio 0.80; 95% confidence interval 0.65–0.98; p = 0.03) and with more strokes or transient ischemic attacks (55 [0.9%] versus 34 [0.5%]; odds ratio 1.56; 95% confidence interval 1.02–2.42, p = 0.04)^12^. In the Chinese population of TOTAL trial, manual thrombus aspiration was significantly associated with a nearly sevenfold increased risk of stroke at 5 years compared with PCI alone^13^. The risk of stroke might be associated with embolization of thrombus from the coronary artery to systemic circulation. AngioJet rheolytic thrombectomy is achieved by injecting pressurized saline through a hypotube by the distal tip of the coronary catheter, thereby leading to a low-pressure zone (Bernoulli effect). Thrombus is fragmented by the saline jets out of the catheter before being evacuated from the body through the catheter. A comparison of manual thrombus aspiration with rheolytic thrombectomy by optical coherence tomography showed that rheolytic thrombectomy group had a lower residual thrombotic burden as compared with manual aspiration^14^. Additionally, the catheter of the AngioJet rhelytic thrombectomy has a lower profile than that of manual thrombus aspiration, which can be more effectively used in tortuous and calcified lesions. Therefore, rheolytic thrombectomy is more effective in thrombus removal than manual aspiration, which might be the main reason why rheolytic thrombectomy did not increase the risk of stroke in our study.

So far, the results of randomized trials on rheolytic thrombectomy in patients with STEMI were controversial. The JETSTENT trial, which enrolled 501 patients with thrombus grade 3 to 5, showed a higher event-free survival rate as compared with direct stenting alone^15^. The MUSTELA trial revealed that thrombectomy had a better post-procedural ST-segment elevation resolution and reduced microvascular obstruction at 3 months in patients with high thrombus load by using Export catheter and AngioJet Ultra catheter in a sequential alternating fashion^16^. On the contrary, the AiMI trial showed increased 1-month mortality and major adverse cardiovascular event (MACE) rate in patients treated with rheolytic thrombectomy as compared with PCI alone^17^. The inconsistencies of these results may be associated with study design, limited sample size, technique, and selection bias of the patients.

Our study showed that AngioJet rheolytic thrombectomy had a significantly lower mortality than the routine treatment group in all patients with TIMI thrombus grade 5. The death rate was still lower in the AT group after propensity-score matching (4.3% vs 6.8%), but the difference was not statistically significant. Notably, there was no statistical differences in the rate of TIMI blood flow grade 3 after PCI between two groups, which indicated that the AngioJet rheolytic thrombectomy failed to improve the TIMI flow condition as compared with routine treatment. As it was a non-randomized retrospect study, there may exist selection bias. The patients underwent AngioJet rheolytic thrombectomy had a higher thrombus burden in real-world experiences since operators won’t take into account thrombus aspiration in patients with mild thrombus burden. The creatine kinase-MB at admission was significantly higher in the AT group, which indicated that the patients of the AT group might have a larger infarct size. Hence, the patients of the AT group might have a more serious clinical condition than the RT group. Patients who are at high risk of adverse outcomes tend to have the maximum benefit from a given treatment, which is called as “quantitative interaction”^18^. A real-world study enrolled 9100 patients diagnosed with STEMI showed that selective aspiration thrombectomy at the operation’s discretion had a comparable mortality rate compared with PCI alone and did not increase the risk of stroke^19^.

Previous studies revealed that patients with high risk might benefit from thrombus aspiration^20,21^. Ruben et al. have reported that rheolytic thrombectomy was associated with a lower target vessel revascularization in patients with acute myocardial infarction complicated by cardiogenic shock^22^. Ahmed et al. found that thrombus aspiration might be associated with improved reperfusion and myocardial salvage especially in STEMI patients presenting after 12 h from symptom onset^23^. Accordingly, further investigations on AngioJet rheolytic thrombectomy among patients with high risk is warranted.

Our study has several limitations. First, this is a non-randomized retrospective study. Although propensity-score matching was used, the baseline characteristics could not be matched utterly. There might be selection bias which influence the findings. Second, our study lack of surrogate markers of myocardial reperfusion, such as myocardial blush grade, ST-segment resolution, or infarct size. Third, the exact door-to-device time had significant missingness and therefore was not analysed in our study. However, we were able to confirm that the percentages of delayed PCI (the time of PCI from symptom onset > 12 h) were comparable between the two groups. Finally, our research was a single-center study with a small sample size. A large multicenter trial is warranted to shed light on the benefit and safety of AngioJet rheolytic thrombectomy in patients with high thrombus burden.

## Conclusion

In patients with Thrombolysis In Myocardial Infarction thrombus grade 5, AngioJet rheolytic thrombectomy did not improve clinical outcomes at 1 year. Meanwhile, AngioJet rheolytic thrombectomy did not increase the risk of stroke in patients with high thrombus burden.
